# Early application of abdominal re-approximation anchor system for the management of the open abdomen

**DOI:** 10.1038/s41598-025-99910-z

**Published:** 2025-05-22

**Authors:** Thamer Nouh, Ahmed Alburakan, Khalil Alawi, Nawaf Alshahwan, Hassan Mashbari, Jalal Alowais, Walid Khalaf

**Affiliations:** 1 Trauma and Acute Care Surgery Unit, Department of Surgery, College of Medicine, King Saud University, Riyadh, Saudi Arabia; 2 Department of Surgery, Faculty of Medicine, Jazan University, Jazan, Saudi Arabia; 3 Department of Surgery, School of Medicine, Al-Imam Muhammad Ibn Saud Islamic University, Riyadh, Saudi Arabia

**Keywords:** Abdominal re-approximation anchor system, Open abdomen, Primary fascial closure, Anatomy, Physics

## Abstract

Managing open abdomens in critically ill patients after damage control surgery is complex and challenging. Delay in definitive surgery reduces successful primary fascial closure increasing complications. Early abdominal wall closure can improve patient outcomes and reduce risks. We retrospectively reviewed all patients who had the abdominal re-approximation anchor applied to achieve primary fascial closure at our institution. The electronic health record was reviewed for demographics, open abdomen indication, total number of operations, time to primary closure, success rate of primary fascial closure, and complications related to the use of the system. Between May 2015 and May 2018, we applied the abdominal re-approximation anchor system to 7 patients managed with open abdomens. Intra-peritoneal sepsis was the indication in 4 patients while the rest were secondary to trauma. The system was applied after an average of 4.7 ± 1.9 days. The fascia was retracted an average of 19 ± 1.5 cm. Tension free fascial closure was achieved in all patients after an average of 5.4 ± 2 days. Early application of the abdominal re-approximation anchor system was associated with achieving tension free delayed primary fascia closure in all our patients with no major abdominal complications.

## Introduction

Damage control surgery with temporary abdominal closure is a common and safe practice used in managing patients with abdominal trauma and severe intra-abdominal sepsis^1,2^. With the understanding of the lethal triad of hypothermia, acidosis, and coagulopathy, the concept of “damage control” emerged, which allowed for abbreviated laparotomy with temporary abdominal closure to stabilize the patients. This was a paradigm shift that prioritized the physiological status of the patient over immediate anatomical repair. Keeping the abdomen open after managing the intra-abdominal emergency decreases the time in which the patient is exposed to coagulopathic stimuli, hypothermia, and acidosis in the operating room and allows initiating a management strategy for proper resuscitation and stabilization in the intensive care unit^3^. A second look allows reassessment and management of intra-abdominal pathology and its complications. The open abdomen is less susceptible to the complications of increased intra-abdominal pressure, such as systemic, respiratory, and organ perfusion issues^4^. Once stabilized, patients are typically returned to the operating room for definitive surgery and abdominal wall closure^1^. Historically, despite significant efforts and supportive measures, these critically ill patients faced high risks of severe complications, including multiple organ dysfunction syndrome (30–40%)^5^, enterocutaneous fistulas (2–25%)^6,7^, intra-abdominal abscesses (83%), and abdominal wall hernias (approximately 25%)^5^. Mortality rates were reported as high as 26%^8^.

Recent registry data have shown promising improvements in outcomes. For instance, one study reported a fascial closure rate of 83.5%, an enteroatmospheric fistula rate of 5.6%, a planned ventral hernia incidence of 6.2%, an in-hospital survival rate of 72%, and an incisional hernia rate of 40.5%. Long-term survival ranged from 22 to 72%^9^. Similarly, an analysis of data from the European Hernia Society (EHS) registry by Willms et al. demonstrated significant reductions in both mortality (from 23 to 14%) and fistula rates (from 17.6 to 5.5%). These improvements were attributed to the rapid direct closure of the fascia, facilitated by a combination of visceral organ protection, negative pressure wound therapy (NPWT), and dynamic fascial traction^10^.

If abdominal closure is not achieved as soon as possible, primary closure of abdominal wall fascia becomes difficult to achieve. Indeed, tension free closure is only accomplished in 51–69% of those patients^11^. This is associated with high risks of complications related to the open abdomen^8,12^. It is recommended that every effort is made to achieve primary facial closure as soon as possible^13^. Different approaches are employed to manage the open abdomen and try to achieve tension free closure of the abdomen to avoid these complications and a real consensus on which technique should be used has not yet been reached^8,14,15^. The introduction of the “Bogota bag” in the 1980s, a simple method using a sterile intravenous fluid bag sutured to the skin to close the abdomen temporarily, marked a significant advancement in temporary abdominal closure techniques^16^. The 1990s saw the development of more sophisticated techniques, including the Wittmann Patch^17^, and vacuum-assisted closure (VAC)^18,19^, which allowed for better fluid management and facilitated delayed primary closure of the abdominal wall. These techniques also improved the management of the open abdomen in septic patients. Component separation techniques were also described in the literature to achieve primary closure in those patients^20^. These approaches achieve abdominal closure with varying success rates but have their own complications such as wound complications and incisional hernias.

Recently, there has seen a focus on developing techniques that allow for dynamic fascial traction, which actively reduces the fascial defect and assists in the eventual closure of the abdomen. Several reports describe the use of dynamic fascial re-approximation in managing the open abdomen^21,22^. The abdominal re-approximation anchor is a method of dynamic fascial traction that has been introduced as an alternative approach to the management of the open abdomen. Its design aims to allow for adjustable tension in re-approximating fascial edges, which is a critical factor when dealing with large or complex abdominal defects. While the system offers a theoretical advantage in mitigating the risks associated with static closure methods, such as compartment syndrome and fistula formation, empirical evidence is still being evaluated. The system’s efficacy in facilitating primary fascial closure and its impact on postoperative morbidity are areas of ongoing research. As the medical community continues to seek optimal strategies for temporary abdominal closure, the abdominal re-approximation anchor’s role remains a subject of investigation. We present our experience with the delayed primary abdominal wall closure via early application using our modified approach of the abdominal re-approximation anchor.

## Materials and methods

### Setting

King Khalid University Hospital is an academic hospital in Riyadh, Saudi Arabia. The hospital has a Trauma and Acute Care Surgery unit that employs attending consultants who are dedicated to managing trauma and emergency general surgery. The experimental protocol was approved by the Institutional Review Board at King Saud University, College of Medicine. All methods were performed in accordance with the relevant guidelines and regulations.

### Patient selection

We included all patients who had a clinical indication to keep their abdomen open and were managed with abdominal re-approximation anchor system application to attempt abdominal closure.

Our practice is to make an assessment, as early as possible after stabilization, mostly at the time of the third look regarding the possibility of achieving primary fascial closure. Once an assessment was made that primary fascial closure was not achievable a decision to apply the abdominal re-approximation anchor is taken to limit further fascia retraction, small bowel adhesion and allow a delayed tension free primary abdominal wall closure. The decision to apply the abdominal re-approximation anchor and the timing of the fascial closure were made by two surgeons from the Trauma and Acute Care Unite to standardize the indication and technique.

### Abdominal re-approximation anchor system application technique

**Fig. 1:**
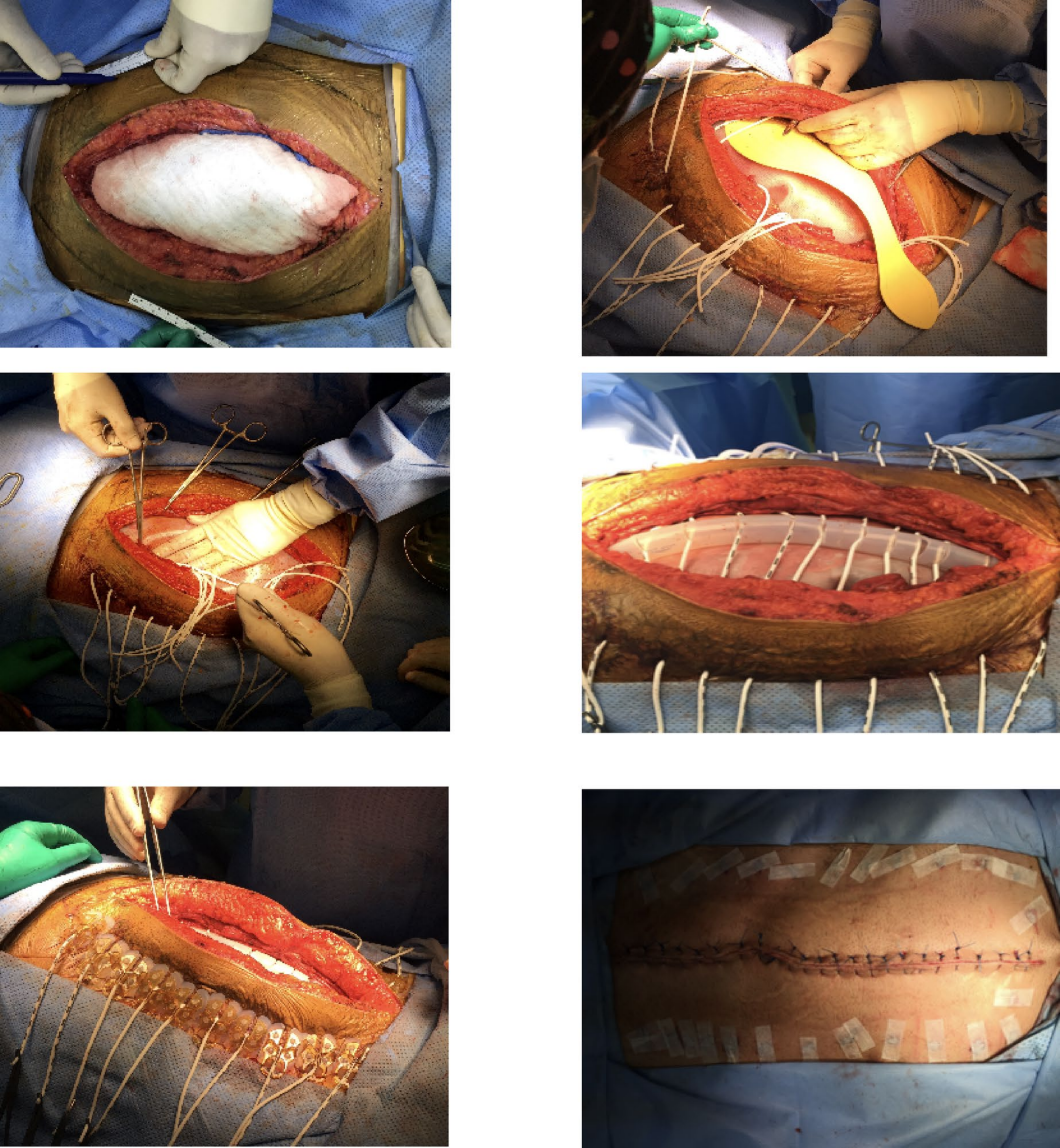
Example of patient with ABRA® application.

**Fig. 2:**
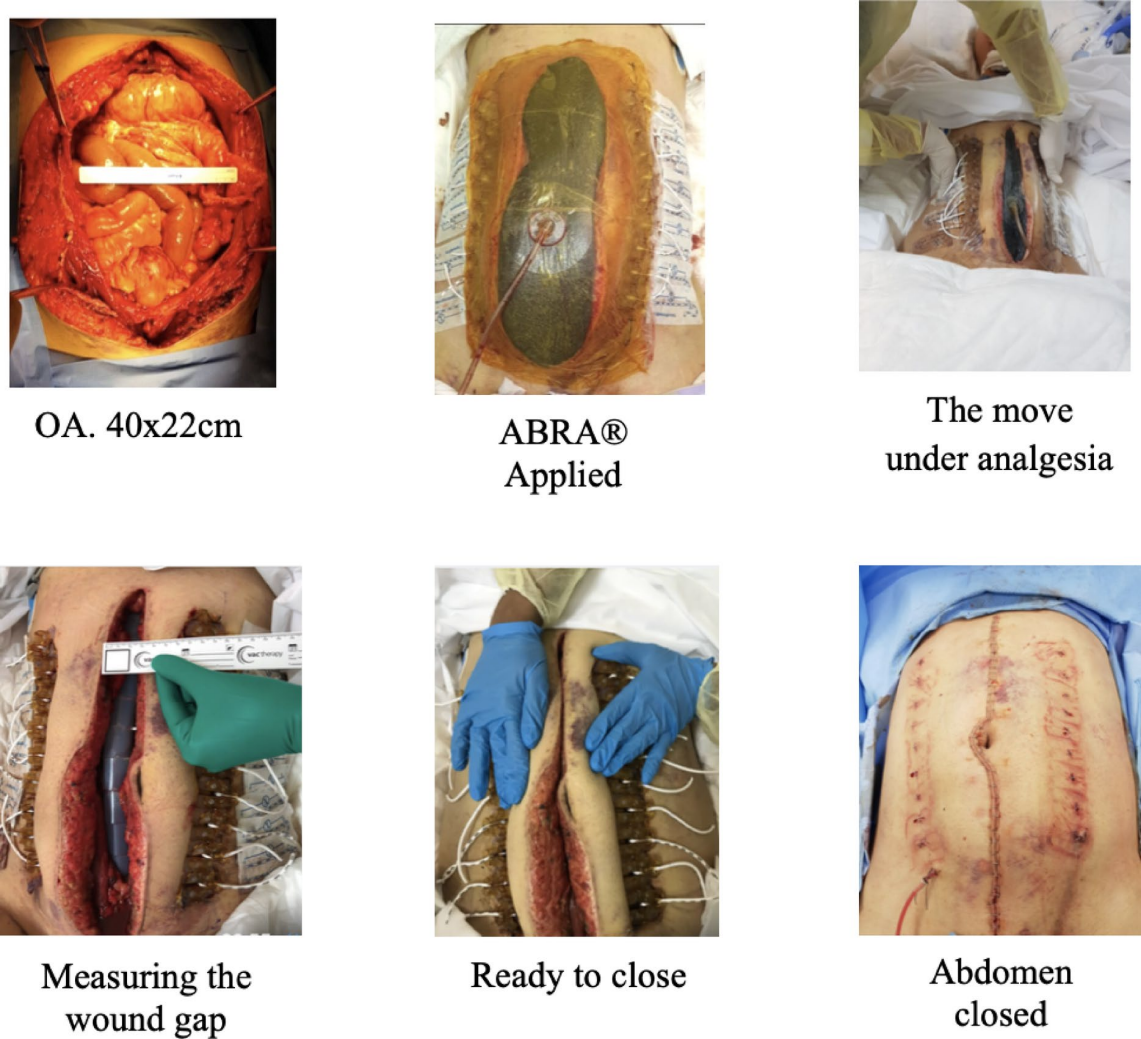
Example of a second patient with ABRA® application.

The abdominal re-approximation anchor system was applied without any debridement to the abdominal wall. Elastomers were inserted through a puncture incision along a line 7 cm lateral to the wound edge, lateral to the edge of rectus sheath and across the defect passing below the fascia over a visceral silicon sheet. The elastomers exit on the contralateral side at an equal distance from the wound edge. The elastomers were placed about 3 cm apart along the wound length, as close as the padding of the button anchors will permit. Button anchors were placed, the elastomers were connected and loaded to about twice their tension marks length, elastomers passed though aligner column keeping the incision aligned in the midline. In addition, to prevent the elastomer tearing medially through the abdominal wall, an adhesive button tail was attached to the anchor and fixed to the skin. Negative pressure wound therapy was applied in all cases (Figs. 1 and 2).

### Temporary dressing technique

Temporary abdominal closure was done using the modified Barker’s method^21^ which consisted of a plastic barrier over the intestines, a burn flap gauze dressing with two suction drains. Ioban was placed over the abdominal wall.

### Post abdominal re-approximation anchor system care

Patients were managed in the intensive care unit in the immediate postoperative care. They were extubated when they no longer required ventilator support. The buttons and skin were cleaned daily. ‘‘The Move’’ as referred to by abdominal re-approximation anchor system manual was delivered by our surgical residents and fellows under supervision of the surgeons. This included massaging the abdominal wall to reshape the abdomen and to mobilize the abdominal wall over the silicone sheet. As the elastomers lost tension they were re-loaded to the maximum tensile strength. When the fascial edges were approximated to a 1 cm distance or less, the patient was taken back to the operating room for standard fascial closure.

### Statistical analysis

The electronic medical records of patients meeting our criteria were reviewed. Detailed demographic and clinical data were collected for each patient (age, gender, body mass index (BMI), acute physiology and chronic health evaluation II (APACHE II) score, open abdomen indication, open abdomen classification according to Bjorck et al.^22^, Mannheim peritonitis index, number of laparotomies to abdominal re-approximation anchor system, time from first laparotomy to abdominal re-approximation anchor system, time from abdominal re-approximation anchor system to facial closure, fluid balance from admission to the application of abdominal re-approximation anchor system, fluid balance from the application of abdominal re-approximation anchor system to abdominal closure, intensive care unit length of stay, hospital length of stay, mortality, abdominal wall complications).

Data were analyzed using descriptive statistics to summarize baseline demographic and clinical characteristics. Continuous variables were expressed as means ± standard deviations. Categorical variables were presented as frequencies and percentages.

## Results

The abdominal re-approximation anchor system was made available at our institution in May 2015. Since its introduction it has been applied to 7 patients managed with open abdomen and managed with this system. Demographics and clinical information are shown in Table 1.

**Table 1:** Characteristics of study patients.

	Age	Gender	Diagnosis	BMI	APACHE II	ICU LOS (Days)	Hospital stay
Traumatic							
1	25	Male	MVC, internal bleeding	27.7	23	13	26
2	30	Female	MVC, internal bleeding	31.1	20	13	86
3	36	Male	Post para-aortic lymph node dissection bleeding	25.3	12	28	57
Abdominal sepsis							
1	14	Female	Pelvic inflammatory mass	30.5	9	28	32
2	37	Male	Obstructed colon cancer	19.4	15	21	65
3	58	Male	Bowel ischemia	24.1	7	28	29
4	73	Male	Obstructed colon cancer	36.9	31	22	70

*BMI* body mass index, *APACHE II* acute physiologic assessment and chronic health evaluation, *LOS* length of stay, *MVC* motor vehicle collision.

The mean age of patients was 39 ± 18.6 years. Most of the patients (n = 5, 71.4%) were men. The average BMI was 27.9 with 71.4% of the patients being overweight or obese. The average APACHE II score was 16.7 ± 7.9. The average intensive care unit length of stay was 25.2 ± 9.9 days, and the average hospital length of stay was 51.97 ± 21.7 days. The indication for open abdomen in our patient cohort was severe intra-peritoneal sepsis (4 patients) and trauma (3 patients).

Our patients had an average Mannheim Peritonitis Index score of 21.9 ± 6.2. According to the Modified Bjorck classifications, there were class 1A (n = 3, 42.8%), class 2A (n = 1, 14.2%) and class 2B (n = 3, 42.8%) open abdomens. The condition of edges of the fascia was good in all patients. Temporary abdominal closure was applied for an average of 4.6 ± 1.8 days before abdominal re-approximation anchor system application. The fascia was retracted an average of 19 ± 1.5 cm at the middle of the wound at the time of abdominal re-approximation anchor system application. The average fluid balance from admission to the time of abdominal re-approximation anchor system application was 5.3 ± 5.0 L.

The “move” was applied daily and the elastomers were adjusted to maintain maximum tensile strength. Tension free closure was accomplished in all 7 patients (100%). The average duration to achieve fully approximated fascia was 5.4 ± 2 days after abdominal re-approximation anchor system application. The average fluid balance from the time of abdominal re-approximation anchor system application to abdominal closure was − 2.2 ± 3.4 L.

**Fig. 3:**
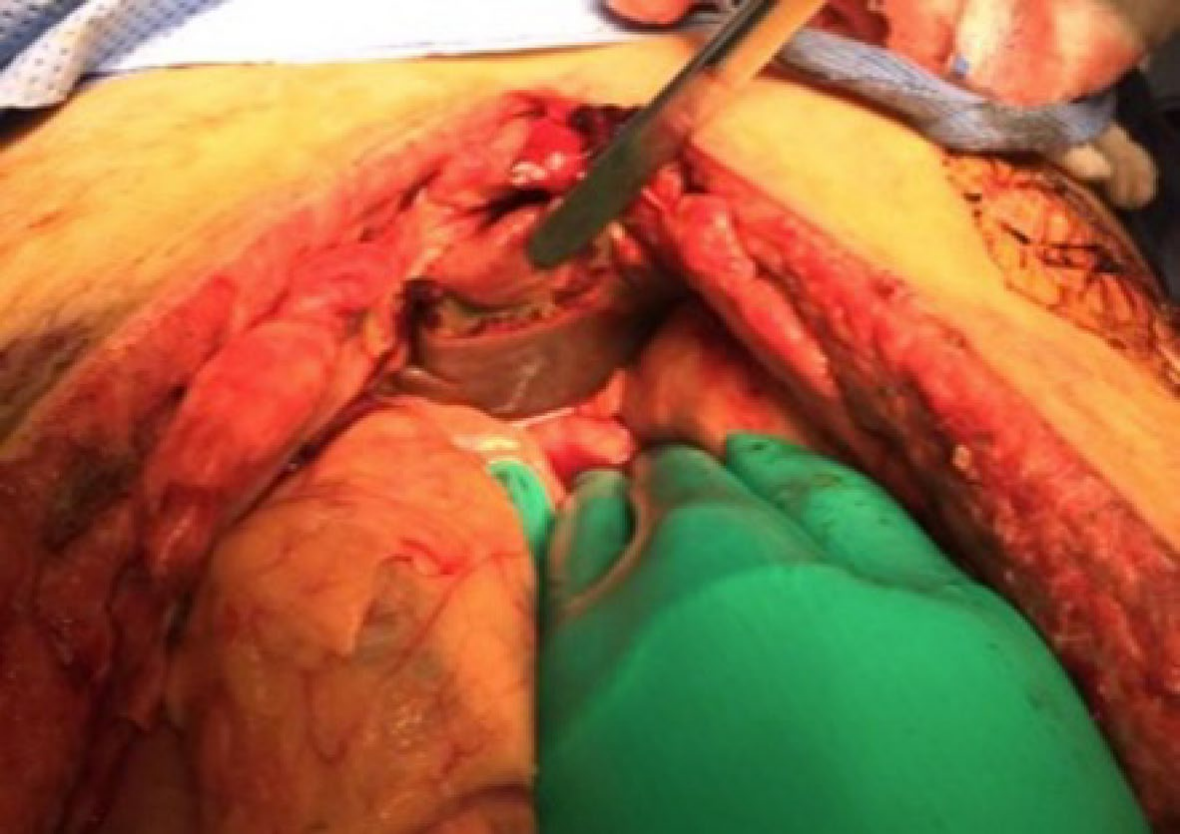
A small liver laceration caused by the elastomers aligning column.

One patient (14.3%) died 3 weeks after a complete abdominal wall closure secondary to severe traumatic brain injury. Two patients (28.6%) developed abdominal re-approximation anchor system related complications. One patient developed a small liver laceration caused by the elastomers aligning column (Fig. 3). The other patient developed a small subcutaneous abscess above an intact closed fascia. For all other patients, there was no skin ulceration at the anchor site, no abdominal wall, or intra-abdominal complications (Table 2).

**Table 2:** Characteristics of study patients’ wounds, ABRA® days, and complications.

	Bjork class	MPI	Length of the wound (cm)	Width of the wound (cm)	TAC-days	Fluid balance admission to ABRA (ml)	Fluid balance ABRA to closure (ml)	ABRA® days	Complications
Traumatic									
1	1A	13	37	18	2	1756.0	− 2297.0	5	None
2	1A	13	35	18	5	14,715.6	2721.2	10	None
3	1A	28	40	21	3	10,088.2	186.0	4	Minor liver injury
Abdominal sepsis									
1	2B	28	30	18	4	4687.1	− 782.9	6	Wound infection
2	2A	21	30	17	6	4695.5	− 5280.0	4	None
3	2B	28	38	21	8	− 1415.0	− 8293.5	5	None
4	2B	22	38	20	4	2806.6	− 1648.5	4	None

*TAC* days, temporary abdominal closure days, *ABRA® Days* number of days with ABRA application, *MPI* Mannheim peritonitis index.

## Discussion

In implementing the principles of damage control surgery within our practice, we have encountered the recurrent challenge of achieving primary fascial closure. A critical endpoint that is often difficult to achieve due to the complex nature of open abdomen cases. Furthermore, the open abdomen is fraught with complications, ranging from infection to fluid imbalance, each posing a significant hurdle in the patient’s recovery journey. At our institution, we have utilized several techniques to manage the open abdomen and progress to abdominal closure including, temporary abdominal closure using the modified Barker’s method^23^, applying the commercial VAC dressing, closing the open abdomen through component separation^20^, utilizing biological mesh^24^, skin grafting with planned hernia, to finally using the abdominal re-approximation anchor system.

In our series, we have achieved a hundred percent tension free fascial closure following open abdomen. This is higher than that reported in the literature^22^. Compared to other reports of abdominal re-approximation anchor system application our approach is different in that we apply the system earlier (4.7 ± 1.9 days), we insert the elastomers at 7 cm from the wound edge (lateral to the rectus border), and we apply the “move’ daily to maintain load on the elastomers and the abdominal wall. This has allowed us to achieve tension free fascial closure sooner (5.4 ± 2 days) in all our patients. Our approach is different from previously reported approaches where the time from first laparotomy to abdominal re-approximation anchor system placement averaged between 7 and 18 days, the elastomers were placed at 5 cm from the wound edge (and most of the time less due to adhesions and a frozen abdomen) with a reported fascial closure rate of 61–100% at an average of 10–62 days after system placement^5,21,22,25–27^.

In our study, counter to the trends commonly reported in existing literature, we observed that the rate of achieving tension-free closure was similar across all etiologies. Notably, septic patients did not exhibit a lower rate of abdominal closure when compared to trauma patients. This finding diverges from established patterns where sepsis is often associated with more challenging closure outcomes. This could be explained by the fact that we did not have any Bjork classification 3 or 4 patients, but we hypothesize that our protocol for the early application of the abdominal re-approximation anchor system may have played a critical role in these outcomes. By intervening early with the abdominal re-approximation anchor system, our approach may have prevented the formation of extensive intra-abdominal adhesions. Adhesions are known to complicate subsequent surgical interventions by creating a ‘frozen abdomen’, where the organs are fixated to the abdominal wall, reducing the pliability of the abdominal cavity. Moreover, the early application of the abdominal re-approximation anchor system may have contributed to the reduced incidence of enterocutaneous fistulas, a complication that not only impedes tension-free closure but also significantly impacts patient morbidity.

Early application of the abdominal re-approximation anchor system also works to counteract the tendency of the abdominal muscles to retract so that one need not wait until the intra-abdominal conditions are ready for closure to apply the system but can apply it with the intention of keeping the abdomen open while preventing further muscle retraction thus saving valuable time that would be lost to re-approximate the retracted muscles. The attempt at using the abdominal re-approximation anchor system to prevent further muscle retraction has been reported before^26^. This time that is saved decreases the rate of developing a frozen abdomen and enterocutaneous fistulas which is directly proportional to the length of time the abdomen is kept open^28,29^.

Despite having a cohort of sick mostly overweight or obese patients with an average Mannheim Peritonitis Index score of 21.9 ± 6.2 only one patient died (14.3%). His death was not related to the open abdomen and its management. Wound complications developed in 2 of the patients (26.8%) which is consistent with other literature reports. We noticed that none of our patients developed any skin breakdown complications which is different from other reports in the literature^5^. This could be explained by the fact that we place the elastomers further away from the wound edge and this allows a wider distribution of force which helps protect the skin.

Our series is limited in that it is not controlled with no comparison to patients managed without the application of the abdominal re-approximation anchor system. The absence of higher modified Bjorck classifications may have influenced the outcomes. This is curtailed by the fact that these patients were managed following the same approach of early application and aggressive approximation. This makes attempts of adoption and reproducibility by other groups feasible. We are also limited by the small number of patients included but given the type of patients and the wide fascial gap in our patient population we would expect at best a primary fascial closure rate equivalent to what is reported in the literature. Although, early application in our patient series was associated with a rate of primary fascial closure of a 100% this is low quality evidence that cannot be used to guide clinical decisions but can only raise questions and generate hypotheses. It would be beneficial to study a bigger volume of patients and multicenter practices.

Based on the search results, there are no specific papers published about utilizing the abdominal re-approximation anchor system in Saudi Arabia. There are few surgeons started adopting the system but still in the process of data collection.

## Conclusions

Early primary fascial closure of the open abdomen has been associated with improved patient outcome. We have utilized it as our first choice to manage patients with open abdomen deemed difficult to close by our surgeons.

Our approach of early application within 3–5 days of the initial laparotomy, the modified technique of inserting elastomers at 7 cm instead of 5 cm from the wound edge, in addition to the aggressive tightening of the elastomers are key to achieve early tension free primary fascial closure.

## Data Availability

The datasets used and/or analyzed during the current study available from the corresponding author on reasonable request.
